# Supplementary material for: Assessing GPT-4 for cell type annotation in single-cell RNA-seq analysis
Source: Nat Methods. 2024 Mar 25;21(8):1462–5. doi: 10.1038/s41592-024-02235-4 (PMC11310073; doi:10.1038/s41592-024-02235-4)
Supplement: Supplementary file 1 [file 41592_2024_2235_MOESM1_ESM.pdf]

nature portfolio

Corresponding author(s): Wenpin Hou, Zhicheng Ji

Last updated by author(s): 11/19/2023

Reporting Summary

Nature Portfolio wishes to improve the reproducibility of the work that we publish. This form provides structure for consistency and transparency in reporting. For further information on Nature Portfolio policies, see our Editorial Policies and the Editorial Policy Checklist.

Statistics

For all statistical analyses, confirm that the following items are present in the figure legend, table legend, main text, or Methods section.

n/a	Confirmed	
☐	☒	The exact sample size (*n*) for each experimental group/condition, given as a discrete number and unit of measurement
☒	☐	A statement on whether measurements were taken from distinct samples or whether the same sample was measured repeatedly
☐	☒	The statistical test(s) used AND whether they are one- or two-sided<br>*Only common tests should be described solely by name; describe more complex techniques in the Methods section.*
☒	☐	A description of all covariates tested
☐	☒	A description of any assumptions or corrections, such as tests of normality and adjustment for multiple comparisons
☐	☒	A full description of the statistical parameters including central tendency (e.g. means) or other basic estimates (e.g. regression coefficient) AND variation (e.g. standard deviation) or associated estimates of uncertainty (e.g. confidence intervals)
☐	☒	For null hypothesis testing, the test statistic (e.g. *F*, *t*, *r*) with confidence intervals, effect sizes, degrees of freedom and *P* value noted<br>*Give P values as exact values whenever suitable.*
☒	☐	For Bayesian analysis, information on the choice of priors and Markov chain Monte Carlo settings
☒	☐	For hierarchical and complex designs, identification of the appropriate level for tests and full reporting of outcomes
☐	☒	Estimates of effect sizes (e.g. Cohen's *d*, Pearson's *r*), indicating how they were calculated

Our web collection on statistics for biologists contains articles on many of the points above.

Software and code

Policy information about availability of computer code

Data collection	The datasets included in this study were all downloaded directly from the original studies that generate the datasets. No software was used for data collection.
Data analysis	Open-source R packages were used to analyze the data in this study. The GPTCelltype package (v.1.0.0) is provided as an open-source software package with a detailed user manual available in Github repository https://github.com/Winnie09/GPTCelltype. The software is released in Zenodo under the accession code DOI: 10.5281/zenodo.8317406 for all versions (https://doi.org/10.5281/zenodo.8317406). SingleR (version 1.4.1), ScType (version 1.0), and CellMarker2.0 were also used for cell type annotation in this study. SingleR was downloaded as an R software package from Bioconductor (https://bioconductor.org/packages/release/bioc/html/SingleR.html). Source code of ScType was obtained from Github (https://github.com/IanevskiAleksandr/sc-type), and an in-house version was implemented for optimized computational efficiency. CellMarker2.0 was accessed directly from the CellMarker2.0 website (http://bio-bigdata.hrbmu.edu.cn/CellMarker/). All codes to reproduce the presented analyses, as well as the in-house version of ScType, are publicly available in Github repository https://github.com/Winnie09/GPTCelltype_Paper and also in Zenodo under the accession code DOI: 10.5281/zenodo.8317410 (https://zenodo.org/record/8317410). R version 4.0.2 was used to perform the analyses in the manuscript.

For manuscripts utilizing custom algorithms or software that are central to the research but not yet described in published literature, software must be made available to editors and reviewers. We strongly encourage code deposition in a community repository (e.g. GitHub). See the Nature Portfolio guidelines for submitting code & software for further information.

Data

Policy information about availability of data

All manuscripts must include a data availability statement. This statement should provide the following information, where applicable:

- Accession codes, unique identifiers, or web links for publicly available datasets
- A description of any restrictions on data availability
- For clinical datasets or third party data, please ensure that the statement adheres to our policy

The data used in this manuscript are all downloaded from publicly available data sources. Specifically, HubMAP Azimuth data were downloaded from the Azimuth website (https://azimuth.hubmapconsortium.org/). GTEx manually annotated cell types and differential gene lists were downloaded from the supplementary materials of the original study. GTEx gene expression matrix was downloaded from the GTEx website (https://gtexportal.org/home/datasets). Marker genes from literature search were downloaded from the supplementary materials of the original study. HCL manually annotated cell types and differential gene lists were downloaded from the supplementary materials of the original study. HCL gene expression matrix was downloaded from figshare (https://figshare.com/articles/dataset/HCL_DGE_Data/7235471). MCA manually annotated cell types and differential gene lists were downloaded from the supplementary materials of the original study. MCA gene expression matrix was downloaded from figshare (https://figshare.com/s/865e694ad06d5857db4b). BCL gene expression matrix and manually annotated cell types were downloaded from Zenodo (https://zenodo.org/record/7813151). Colon cancer gene expression matrix and manually annotated cell types were downloaded from GEO under accession number GSE132465. Lung cancer gene expression matrix and manually annotated cell types were downloaded from GEO under accession number GSE131907. TS gene expression matrix and manually annotated cell types were downloaded from UCSC Cell Browser(https://cells.ucsc.edu/?ds=tabula-sapiens). Marker genes and cell type annotations for the non-model mammal dataset were downloaded from the supplementary materials of the original study. All relevant information about data is described in the Methods section. All data generated in this study are included in the supplementary table

Human research participants

Policy information about studies involving human research participants and Sex and Gender in Research.

Reporting on sex and gender	NA
Population characteristics	NA
Recruitment	NA
Ethics oversight	NA

Note that full information on the approval of the study protocol must also be provided in the manuscript.

Field-specific reporting

Please select the one below that is the best fit for your research. If you are not sure, read the appropriate sections before making your selection.

☒ Life sciences ☐ Behavioural & social sciences ☐ Ecological, evolutionary & environmental sciences

For a reference copy of the document with all sections, see nature.com/documents/nr-reporting-summary-flat.pdf

Life sciences study design

All studies must disclose on these points even when the disclosure is negative.

Sample size	A total of 10 datasets were included in the study. The Azimuth study contains 11 tissues and 276 cell types. The Human Cell Atlas (HCA) study contains 7 tissues and 72 cell types. The Human Cell Landscape (HCL) study contains 60 tissues and 101 cell types. The literature search dataset contains 7 tissues and 30 cell types. The Mouse Cell Atlas (MCA) study contains 51 tissues and 65 cell types. The lung cancer and colon cancer tissue contain 10 and 7 cell types, respectively. Non-model mammal contains 5 tissue and 24 cell types. GTEx dataset contains 7 tissue and 41 cell types. Tabula sapiens dataset contains 24 tissue and 171 cell types. B cell lymphoma (BCL) dataset contains one tissue and 9 cell types. The number of datasets was determined by the available datasets we can identify that represent a wide variety of tissues. The number of dataset is already sufficiently large since these datasets already represent a wide variety of tissues, cell types, and species so that the conclusions in this study is reliable enough.
Data exclusions	For the literature search dataset, only cell types with at least 5 marker genes were used. For other four datasets, all cell types included in the original studies were tested and evaluated in this study.
Replication	All simulation studies were repeated five times in this study.

Randomization	This study depends on publicly available data that were already collected and published by other research groups. Randomization is not applicable since no new web-lab experiment is conducted in this study.
Blinding	Blinding is not applicable since there is no clinical research data involved in this study.

Reporting for specific materials, systems and methods

We require information from authors about some types of materials, experimental systems and methods used in many studies. Here, indicate whether each material, system or method listed is relevant to your study. If you are not sure if a list item applies to your research, read the appropriate section before selecting a response.

Materials & experimental systems

n/a	Involved in the study
☒	☐ Antibodies
☒	☐ Eukaryotic cell lines
☒	☐ Palaeontology and archaeology
☒	☐ Animals and other organisms
☒	☐ Clinical data
☒	☐ Dual use research of concern

Methods

n/a	Involved in the study
☒	☐ ChIP-seq
☒	☐ Flow cytometry
☒	☐ MRI-based neuroimaging
